# Supplementary material for: Contextual experience modifies functional connectome indices of topological strength and efficiency
Source: Sci Rep. 2020 Nov 16;10:19843. doi: 10.1038/s41598-020-76935-0 (PMC7670469; doi:10.1038/s41598-020-76935-0)
Supplement: Supplementary file 1 — Supplementary Information. [file 41598_2020_76935_MOESM1_ESM.docx]

**Contextual experience modifies functional connectome indices of topological strength and efficiency**

Marjory Pompilus1,2, Luis M. Colon-Perez^4^, Matteo M. Grudny1, Marcelo Febo1,2,3

1 Department of Psychiatry, ^2^Advanced Magnetic Resonance Imaging and Spectroscopy (AMRIS) Facility, ^3^Evelyn F. and William L. McKnight Brain Institute, College of Medicine, University of Florida, Gainesville, Florida; ^4^Center for the Neurobiology of Learning and Memory, Department of Neurobiology and Behavior, School of Medicine, University of California, Irvine, California


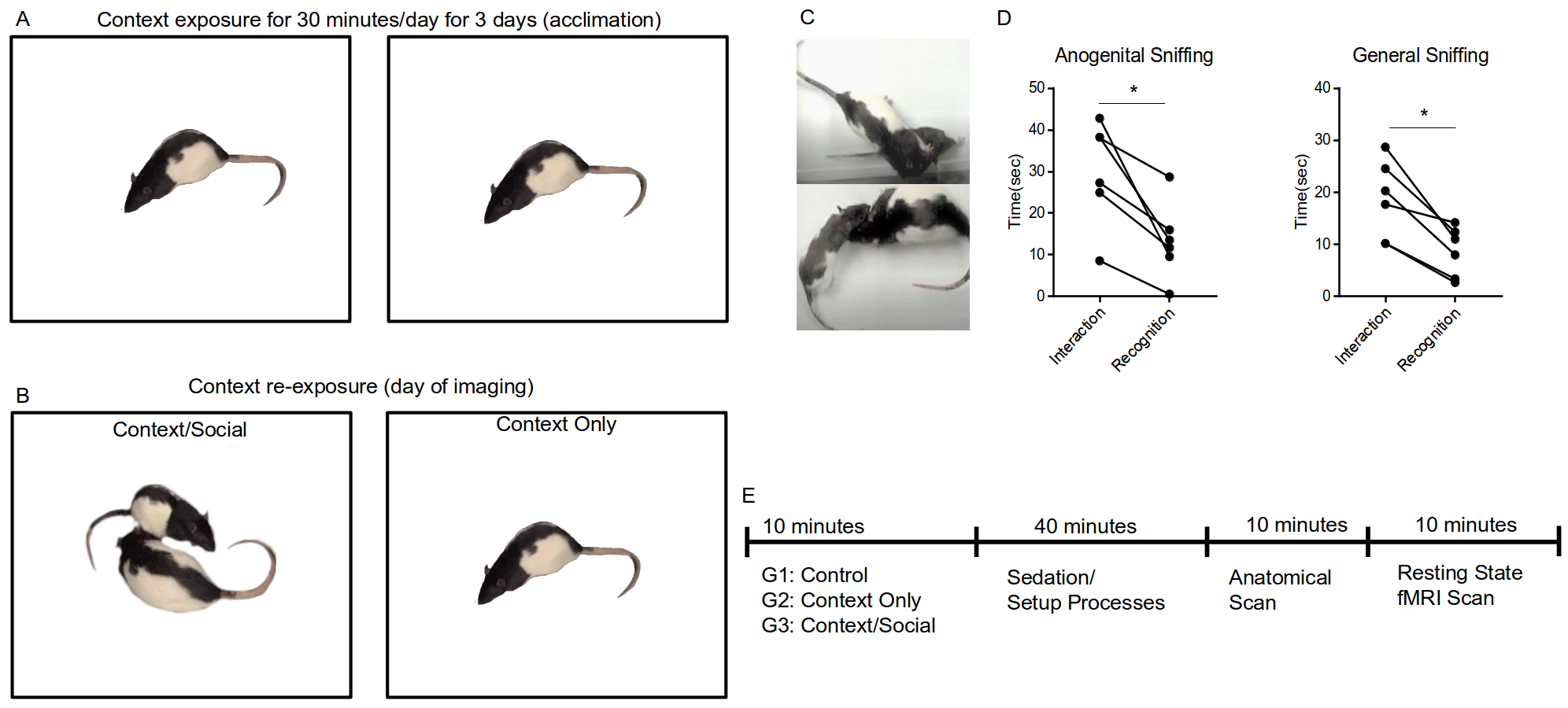
**Supplementary Figure 1.** **Timing of pre-scan stimulus presentation, sedation, setup, and image acquisition were kept the same across all subjects.** A) Rats are placed into the same test cage over 3 days prior to imaging (become familiar). B) Before imaging, rats are once again presented with the familiar context either in the absence or presence of an unfamiliar social stimulus (juvenile rat). C) Digital video frames showing visually confirmed social interactions. D) Assessments of exploratory sniffing. Rats explore novel juvenile rats at a much higher level at the first 10 minute test than in a second test 1 hour later (paired t-test, p<0.05). E) Temporal sequence between stimulus presentation and fMRI acquisition.

**
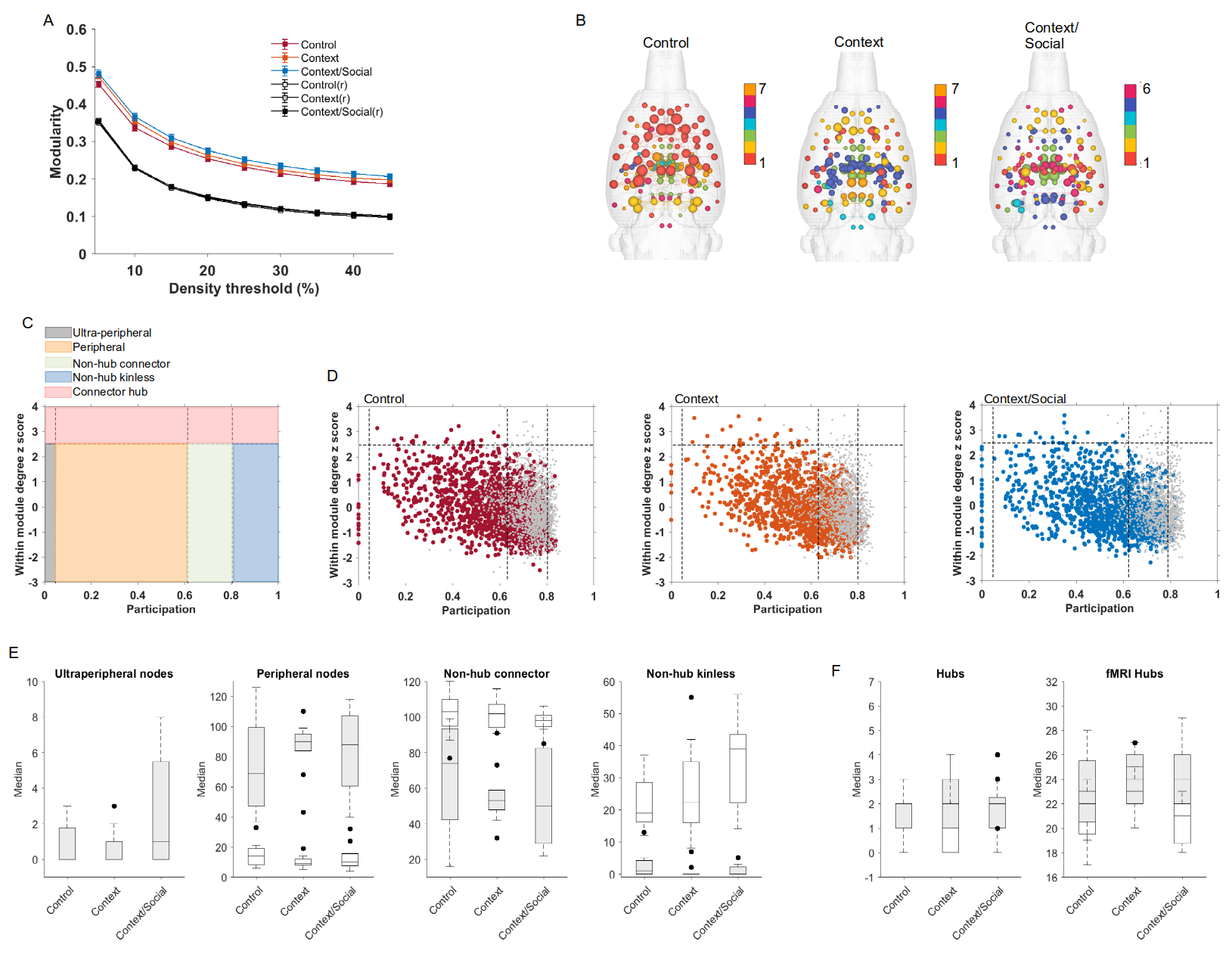
**

**Supplementary Figure 2. Context and context/social exposure did not alter modular organization of nodes.** A) Modularity index at various densities. B) 3D connectome maps with nodes scaled by modularity index Q in control, context exposed, and context/social exposed. Scale bar color represent community affiliation of nodes (network density at 10%). C) Nodal assignment chart according to Guimerà and Amaral ^1^. D) Scatter plots of within module degree z score versus participation coefficients per node for all subjects in control (red), context (orange) and context/social (blue) exposed rats. Hashed lines represent limits for specific ranges for cartographic assignments per node according to ^1^ . E) Nodal role assignments according to participation coefficient values. F) Nodal assignments according to within module degree z scores (hubs > 2.5; fMRI hubs > 1 ^2^). Network density for data in D-F were calculated for a network density of 10%. In A, blue, orange, red line plots are for randomized (r) versions of functional connectivity matrices. In D, gray color dots represent data points for nodes or edges of randomized graphs. In E-F, data are median ± 95% confidence intervals (box-whisker plots, with black dots representing outliers). Gray boxes are for real fMRI networks and empty are for randomized versions of the same networks. Data are mean ± standard error. *Context and **context/social group significantly different from control (ANOVA with Tukey-Kramer post hoc test).


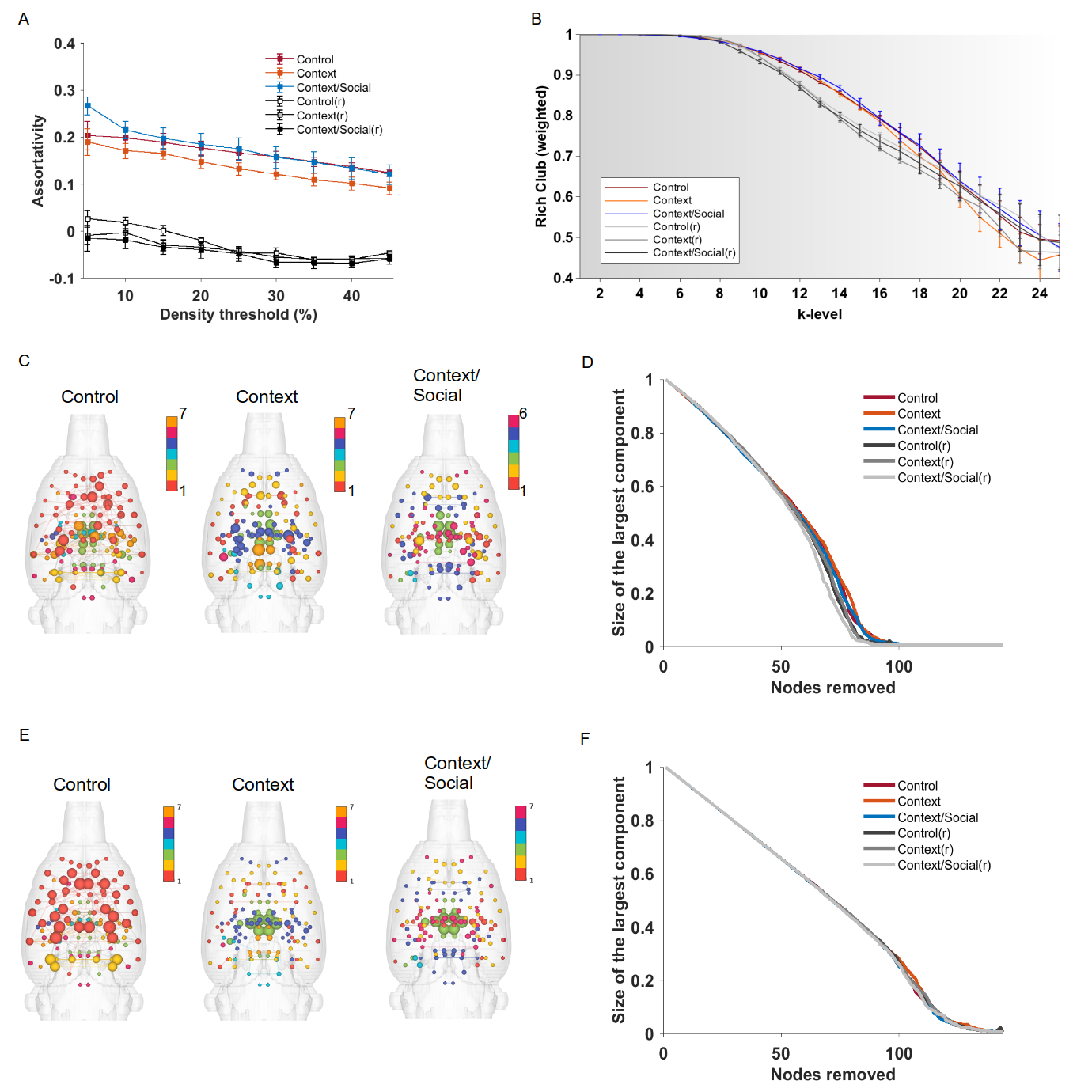
**Supplementary Figure 3. Context and context/social exposures did not alter functional connectivity between strongly weighted nodes and did not alter network robustness.** A) Assortative mixing at various graph densities. B) Rich club curve for a 10% graph density. C) 3D connectome maps with nodes scaled by betweenness centrality ranking in control, context exposed, and context/social exposed. Scale bar color represent community affiliation of nodes. D) Size of the largest component as a function of node removal according to descending betweenness centrality ranking. E) 3D connectome maps with nodes scaled by eigenvector centrality ranking in control, context exposed, and context/social exposed. Scale bar color represent community affiliation of nodes. F) Size of the largest component as a function of node removal according to descending eigenvector centrality ranking. Graph density for D-F is 10%. Data in A-B are mean ± standard error. In A, blue, orange, red line plots are for randomized (r) versions of functional connectivity matrices. In B, yellow-orange line plots are for randomized (r) data sets. In D and F, gray scale colored curves are for randomized (r) data sets.

**
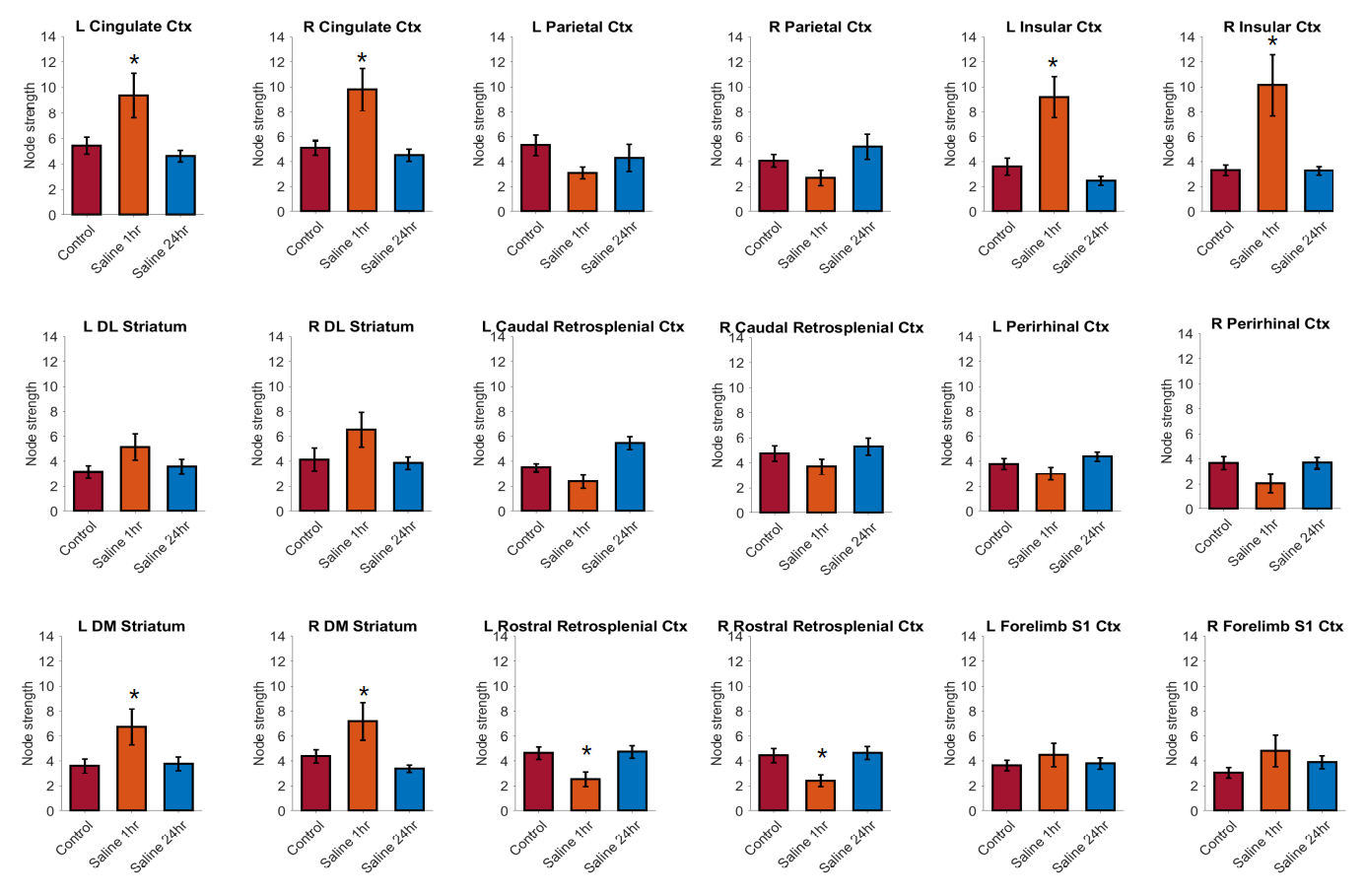
**

**Supplementary Figure 4. Differential effects of the arousing stimulus on local node strength in regions also assessed in response to context and social/context exposures (Figure 2).** Data are mean ± standard error. Node strength calculated for a graph density of 10%. *Context and **context/social group significantly different from control (ANOVA with Tukey-Kramer post hoc test). Abbreviations: DM, dorsomedial; DL, dorsolateral; L, left; R, right; Ctx, cortex; S1, primary somatosensory.

**Supplementary Table 1.** Areas that had bilateral changes in node strength in response to an arousing pre-scan stimilus (saline injection 10 minutes prior to imaging setup). One way ANOVA results (p values FDR corrected across 144 regions)

| Region | Statistical result (by hemisphere) |
| --- | --- |
| Anterior cingulate cortex | left: F_2,21_=6.4, p=0.01; right: F_2,21_=9.1, p=0.004 |
| Insular cortex | left: F_2,21_=14.1, p=0.001; right: F_2,21_=10.9, p=0.002 |
| Dorsomedial striatum | left: F_2,21_=4.4, p=0.03; right: F_2,21_=5.5, p=0.02 |
| Rostral retrosplenial cortex | left: F_2,21_=4.6, p=0.02; right: F_2,21_=4.3, p=0.03 |

**Supplementary Table 2.** Additional areas that had changes in node strength in response to an arousing pre-scan stimilus (saline injection 10 minutes prior to imaging setup). One way ANOVA results (p values FDR corrected across 144 regions)

| Region | Statistical Result (by hemisphere) |
| --- | --- |
| Anterior amygdala  Basal amygdala  Central amygdala  Lateral amygdala  Medial amygdala  Bed nucleus of the stria terminalis  Prelimbic cortex  Primary motor cortex  Secondary motor cortex  Caudal piriform cortex  Lateral septal nucleus  Medial preoptic area  Central grey  Nucleus accumbens  Ventromedial striatum  Ventral pallidum | left: F_2,21_=6.5, p=0.009; right: F_2,21_=5.1, p=0.02 left: F_2,21_=15.7, p=0.0001; right: F_2,21_=13.3, p=0.001  left: F_2,21_=4.8, p=0.02; right: F_2,21_=14.3, p=0.001  left: F_2,21_=11.2, p=0.002; right: F_2,21_=14.0, p=0.0009  left: F_2,21_=9.0, p=0.004; right: F_2,21_=16.8, p=0.001  left: F_2,21_=5.6, p=0.02; right: F_2,21_=4.7, p=0.02  left: F_2,21_=3.7, p=0.04; right: F_2,21_=10.5, p=0.002  left: F_2,21_=8.4, p=0.004; right: F_2,21_=11.8, p=0.002  left: F_2,21_=4.0, p=0.03; right: F_2,21_=3.6, p=0.04  left: F_2,21_=5.1, p=0.02; right: F_2,21_=8.9, p=0.003  left: F_2,21_=10.6, p=0.002; right: F_2,21_=12.5, p=0.001  left: F_2,21_=7.8, p=0.005; right: F_2,21_=6.7, p=0.008  left: F_2,21_=8.5, p=0.004; right: F_2,21_=7.3, p=0.007  left: F_2,21_=6.5, p=0.009; right: F_2,21_=7.2, p=0.007  left: F_2,21_=15.1, p=0.0001; right: F_2,21_=29.6, p<0.00001  left: F_2,21_=13.1, p=0.001; right: F_2,21_=7.4, p=0.006 |

**CITED REFERENCES**

1. Guimerà, R. & Amaral, L. A. N. Cartography of complex networks: Modules and universal roles. *Journal of Statistical Mechanics: Theory and Experiment* **2005**, 1–13 (2005).

2. Meunier, D., Achard, S., Morcom, A. & Bullmore, E. Age-related changes in modular organization of human brain functional networks. *NeuroImage* **44**, 715–723 (2009).
